# Tenascin-X—Discovery and Early Research

**DOI:** 10.3389/fimmu.2020.612497

**Published:** 2021-01-11

**Authors:** Walter L. Miller

**Affiliations:** Department of Pediatrics, Center for Reproductive Sciences, and Institute of Human Genetics, University of California, San Francisco, CA, United States

**Keywords:** adrenal, 21-hydroxylase, human leukocyte antigen locus, extracellular matrix, Ehlers-Danlos syndrome, major histocompatibility locus, CYP21, cytochrome P450

## Introduction

Tenascin-X (TNX) is a large extracellular matrix protein discovered because its *TNXB* gene overlaps the *CYP21A2* gene encoding steroid 21-hydroxylase (P450c21), whose mutations cause congenital adrenal hyperplasia (CAH). In the 1980s, several laboratories worked to clone the “CAH gene”. We sought a P450c21 clone in a cDNA library prepared from a CAH adrenal and identified a transcript larger than P450c21 cDNA; sequencing indicated it was encoded by an unknown (”X“) gene overlapping *CYP21A2* on the opposite DNA strand. Extensive genomic sequencing revealed the structure of a tenascin: N-terminal EGF-like repeats, multiple fibronectin-III repeats, and a C-terminal fibrinogen-like domain; we named this “Tenascin-X” (TNX). To study TNX function, we postulated a “contiguous gene syndrome”—a single mutation affecting both *CYP21A2* and *TNXB*, causing CAH plus another disorder that might suggest the role of TNX. A patient with CAH and Ehlers-Danlos syndrome (EDS) had partial deletions encompassing both genes. With collaborators, we described patients with recessive TNX-deficient EDS (now termed “classic-like EDS”), which was clinically distinct from dominant EDS caused by collagen mutations. TNX haploinsufficiency causes the mild “hypermobility form” of EDS, often associated with CAH, comprising the unique CAH-X syndrome. The discovery of TNX illustrates scientific serendipity and the value of pursuing unexpected results.

## Stumbling Onto “Gene X”—An Unexpected Finding in an Endocrine Study

**Figure 1:**
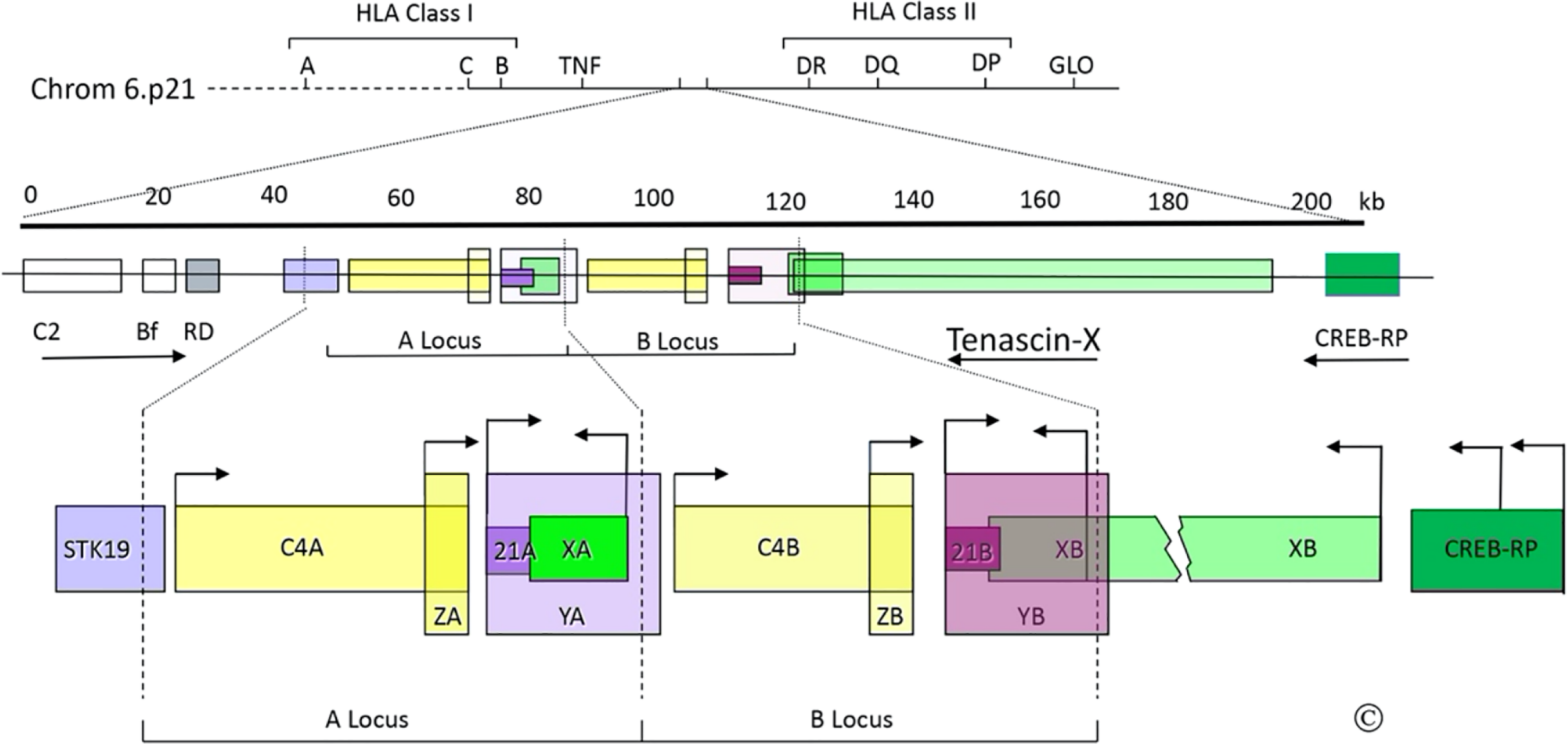
The C4/CYP21/TNX gene locus. Top: Diagram of the short arm of chromosome 6; the telomere is to the left and the centromere is to the right. The MHC Class I and Class II regions are indicated with their principal human leukocyte antigen (HLA) genes; the ~1 megabase region between these is the “Class III region”, which includes the gene for tumor necrosis factor (TNF). Middle: Scale bar in kilobases (kb) and enlarged view of a portion of the Class III region (chrom 6p21.33); the arrows indicate transcriptional orientations. C2, complement factor C2; Bf, properedin factor Bf; RD is now known as NEFLE, negative elongation factor subunit E; CREB-RP, CREB-related protein. Bottom: The duplicated 30 kb C4/CYP21/TNX units and adjacent regions: STK19, serine/threonine kinase 19; C4A and C4B, genes for complement component 4; 21A, *CYP21A1P* pseudogene; 21B *CYP21A2* gene; XA, YA, and YB, adrenal transcripts that lack open reading frames; XB, the *TNXB* gene; XB-S the short, adrenal-specific form of TNX, arises from the leftward transcription arrow within the XB gene, analogously to XA; ZA and ZB, adrenal-specific transcripts with open reading frames arising from promoters within the C4 genes; the ZA and ZB promoters are enhancer elements of the *CYP21A1P* and *CYP21A2* promoters. Most TNX transcription arises from the untranslated exon at the 5’end of *TNXB*, but some also arises from two sites within CREB-RP. The vertical dotted lines designate the boundaries of the gene duplication event. © WL Miller.

The discovery of TNX and its associated deficiency disease (a form of Ehlers-Danlos Syndrome) was done in studies of human steroidogenesis and its disorders rather than in studies of connective tissues. In the 1980s, application of then-new molecular biologic techniques revolutionized understanding of steroidogenesis (1). A driving force behind this early work was steroid 21-hydroxylase deficiency (21OHD), a form of congenital adrenal hyperplasia (CAH) causing cortisol deficiency, potentially-lethal aldosterone deficiency, and androgen excess with prenatal virilization of affected females. There are many forms of CAH, but 21OHD, with an incidence of ~1:15,000 (2) was responsible for >90% of cases; 21OHD is now well understood, but requires intensive management (3). Adrenal 21-hydroxylation is catalyzed by P450c21 (CYP21), a microsomal cytochrome P450 enzyme. In 1986, we reported the bovine *Cyp21* gene sequence (4) and others (5, 6) reported the human gene. The gene lay in the human leukocyte antigen (HLA) locus (major histocompatibility locus, MHC) on chromosome 6p21.33, the most gene-dense and highly recombinogenic region of the human genome. Duplicated 30-kb units contained the functional *CYP21A2* gene and a non-functional *CYP21A1P* pseudogene duplicated in tandem with the *C4A* and *C4B* genes encoding the fourth component of serum complement (7–9) (**Figure 1**). *CYP21A1P* is transcribed (10, 11), but is considered a pseudogene because its RNAs do not encode protein. Human *CYP21A2* encodes P450c21, in mice the *cyp21a1* gene corresponding to *CYP21A1P* is active (12, 13), in cattle both genes function (4, 14), and some other mammals have single copies of this locus (15); thus the gene duplication post-dates mammalian speciation (16).

There was great interest in studying *CYP21* genes in patients with 21OHD. We obtained adrenal mRNA from an abortus with 21OHD, prepared a cDNA library, and screened it with radiolabeled double-stranded P450c21 cDNA to obtain the cDNA for the responsible mutant gene (17). Both the known cDNA sequence and RNA blotting showed that the full-length cDNA would be 2.0 kb, but several clones were longer than that, suggesting that the 21OHD might have arisen by an RNA splicing error. Restriction endonuclease mapping of the longest (2.7 kb) clone indicated that it contained only the 3’ end of *CYP21*. A 2.7 kb clone might have been a cloning artifact, a recombination between *CYP21* and something else (as sometimes happened with the reagents then available), but because we had screened the cDNA library with a double-stranded probe, we considered that the 2.7 kb clone could have arisen from a transcript on the opposite strand of DNA from the *CYP21* genes. DNA sequencing showed that the 2.7 kb clone had a 3’ poly(A) tail with upstream DNA that matched the predicted opposite-strand sequence of final exon of *CYP21*. The complete 2.7 kb sequence showed an open reading frame with repeating units whose structural significance was not clear (17). Because the CYP21/C4 locus is duplicated, we knew that this new “gene X” must also be duplicated as “XA” and “XB” genes with the arrangement 5’-C4A-21A-XA-C4B-21B-XB-3’ (**Figure 1**). Deletions in the gene causing 21OHD did not appear to extend into the XB gene, but deletions of the XA region were found in 14% of human chromosomes (18, 19), suggesting that the 2.7-kb cDNA arose from the XB gene (17). But the nature of the gene encoding the 2.7-kb cDNA was unknown—the operational name “gene X” thus became the source of the name “Tenascin-X”.

## Gene X Becomes Tenascin-X

The publication of the 2.7 kb cDNA led us and others to study its gene structure. In 1989, Dr. Russell Doolittle (UC-San Diego), told us that the sequence of our 2.7 kb cDNA resembled chicken tenascin, and published a paper describing a fibrinogen-like sequence in an invertebrate, saying “The sea cucumber protein also corresponds exactly with a segment found as part of the previously unidentified gene product found in human adrenals” (20). Our 2.7 kb cDNA sequence had domains for fibrinogen and fibronectin type III (Fn-III) repeats, thus resembling tenascin (16). Matsumoto et al. confirmed the identification of fibrinogen and Fn-III domains, identified tenascin-like EGF-like domains and provided additional information about exonic organization (21, 22). To determine how this locus was duplicated, we sequenced genomic DNA at the predicted duplication boundaries and through the entire 7 kb between 21A and C4B that had to comprise the XA locus, providing the entire XA gene sequence and the precise boundaries of the human gene duplication (16). These boundaries were substantially different from the corresponding duplication loci in the mouse genome, as expected for independent duplication events that post-dated mammalian speciation. Although XA was abundantly expressed in the adrenal, its gene was truncated at its 5’ end (compared to XB) by the gene duplication (16), suggesting that it is a pseudogene and that XB would be the more important locus.

Manual sequencing of overlapping genomic clones revealed the nearly complete structure of the XB gene: 39 exons spanning 65 kb encoding a protein of >400-kDa (23). Some Fn-III repeats underwent alternative splicing; current data show 44 exons spanning 68 kb encoding 4,244 amino acids totaling 458,220 Da (24). The structure contained the five domains expected of a tenascin. First, the N-terminus comprised a 22AA signal peptide that directs the protein to the secretory pathway, used by extracellular matrix proteins. Second is a hydrophobic domain containing three heptad repeats that encode the tenascin “head piece”, which permits polymerization of tenascin monomers into multi-armed “brachion” structures. The three heptad repeats suggested that TNX should form a “tri-brachion”, similar to the hexabrachion structure of chicken (25) and human (26) tenascin. TNX lacked the additional cysteine residues in this domain of tenascin and restrictin, which permit two tri-brachions to pair into a hexabrachion. The tri-brachion structure of TNX was subsequently confirmed (27); with glycosylation, a TNX tri-brachion is ~1.5 million Da. Third, a single exon encoded a series of 18.5 EGF-like repeats having 55% similarity to the 13.5 EGF-like repeats of human tenascin/cytotactin. Fourth, a series of evolutionarily duplicated exons encoded 32 Fn-III repeats, including the cell-binding domain identified in chicken tenascin (25). Finally, the last five exons encode the carboxy-terminal fibrinogen-like domain and the 3’-untranslated region, including the domains that overlap *CYP21A2* (17). The carboxy-terminal fibrinogen-like domain was widely conserved in evolution (20, 28), and the sequence and intron/exon arrangement of the 3’ end of XB were very similar to the β- and γ- chains of fibrinogen (29). Thus, the product of the XB gene was a member of the family of tenascins. We said “We suggest that this category of proteins be termed ‘brachions’ or ‘tenascins’. We favor the latter. Tenascin, the first-described member, which is also widely termed ‘cytotactin’, would be termed tenascin-C or TN-C to designate tenascin-cytotactin; restrictin would be termed tenascin-R or TN-R; and the product of the XB gene described in this paper would be tenascin-X or TN-X. This system would emphasize the relatedness among the monomeric units of these proteins and would, to the extent possible, incorporate terminologies and letterings favored by various groups. It seems unlikely that the number of tenascins will exceed the confines of the alphabet.” (23). Thus, the currently used nomenclature for the tenascins, TNC, TNR, and TNX, was established, soon to be followed by TNW (30, 31).

## Additional Studies and Genes in the *TNX* Gene Locus

The structure of TNX is conserved in mice, with subunits of ~500 kD expressed in a pattern distinct from TNC (32). Expression of *TNXB* is tissue-specific and developmentally regulated (33, 34). In fetal adrenal, fetal muscle, and skin HT1080 cells, *TNXB* transcription begins with an untranslated exon ~10 kb upstream from the first coding exon (35). The *CREB-RP* gene encoding the transcription factor CREB-related protein lies immediately upstream from *TNXB* (36). *TNXB* transcripts arise from multiple Sp1/Sp3 sites near to and within *CREB-BP* (35, 37); thus, both ends of *TNXB* overlap other genes (**Figure 1**). Because *XA* is transcribed despite lacking promoter sequences comparable to those of *TNXB*, we characterized the 128 bp *XA* promoter lying between *XA* and *C4B* (38). This sequence is identical in *TNXB* and drives the adrenal-specific expression of a truncated 74 kDa form of TNX, called XB-Short (XB-S), which is identical to the carboxy-terminal 673 amino acids of TNX (38) (**Figure 1**). Expression of XB-S is induced by hypoxia (39), and XB-S associates with mitotic motor kinesin Eg5 (40), but its precise function remains unclear. Additional transcripts termed YA and YB arise from the *CYP21A1P* and *CYP21A2* promoters, but do not encode protein (10), and transcripts termed ZA and ZB arise from a promoter element within intron 35 of the *C4* genes, but it is not clear whether these open reading frames encode protein (41). The ZB promoter is an upstream adrenal enhancer element for *CYP21A2* (42). The location of this essential *CYP21A2* element within *C4B* (also seen in the mouse) (43), explains why the *C4*, *CYP21*, and *TNX* genes remain intimately linked in mammalian genomes.

## Tenascin-X Deficiency Causes an Autosomal Recessive Form of Ehlers-Danlos Syndrome

Developmental expression of *TNXB* showed a recurring pattern, appearing first in connective tissue surrounding muscle and then in a subset of intramuscular cells, suggesting roles in muscle morphogenesis (33). To find a biological role for TNX, we hypothesized that an HLA-linked deficiency disease for TNX might exist, but no clinical candidates emerged. Another approach was to postulate existence of a “contiguous gene syndrome” comprising a partial deletion of both the *CYP21A2* and *TNXB* genes, so we sought a patient with 21OHD “and something else”. Serendipitously, Dr. Cynthia Curry (Fresno CA), asked us about a patient with 21OHD and a connective tissue disorder that resembled Ehlers-Danlos Syndrome (EDS). EDS was then known as an autosomal dominant disorder of collagen deposition, with rare recessive forms in collagen-modifying enzymes, (lysyl hydroxylase or pro-collagen N-proteinase) (44), hence TNX was not an obvious candidate. The patient’s skin had ultrastructural findings atypical for known forms of EDS. An antiserum that recognized multiple TNX epitopes detected TNX in cultured dermal fibroblasts from controls and from the obligately-heterozygous parents, but not in the patient’s fibroblasts; similarly, control, but not patient fibroblasts contained TNX mRNA, confirming TNX-deficiency. Because the protein-coding regions of *CYP21A2* and *TNXB* do not overlap, we sought gene deletions rather than point mutations. Genomic PCR and Southern blotting identified a deletion extending from *XA* through *CYP21A2* to the corresponding point in *TNXB*, demonstrating that TNX deficiency causes EDS (45). In collaboration with Prof. Joost Schalkwijk (U. Nijmegen, Netherlands), we found an immunoassayable TNX fragment in the sera of 146 of 151 patients with EDS; the five patients lacking serum TNX had *TNXB* mutations, none of which encompassed *CYP21A2* (46). Subsequent work has confirmed that TNX deficiency causes a clinically distinct, severe form of EDS (47, 48). Similarly, *Tnxb*-knockout mice had skin hyperextensibility, reduced skin tensile strength and reduced skin collagen content (49); whereas mouse knockouts of TNC and TNR lacked abnormal phenotypes (50–52). TNX appears to associate with and stabilize newly produced collagen fibrils (27, 53, 54), thus all recessive forms of EDS concern post-translational modification of collagens.

## Congenital Adrenal Hyperplasia and Tenascin-X—The CAH-X Syndrome

TNX has functions beyond EDS (55); it promotes epithelial-mysenchymal transitions in development (56), and may be associated with tumor invasion (57–59). TNX-deficiency has been associated with primary myopathy (60, 61), recurrent gastrointestinal perforation (62), and vesicoureteral reflux (63, 64). TNX is expressed in leptomeninges and choroid plexus (34, 65), suggesting neurologic roles: *TNXB* single nucleotide polymorphisms are associated with schizophrenia (66, 67), and *Tnxb*-knockout mice have increased anxiety, improved memory, and higher sensorimotor coordination than controls (68).

While TNX-deficient EDS is autosomal recessive, heterozygous *TNXB* mutations cause TNX haploinsufficiency, with joint hypermobility, recurring joint dislocations and joint pain—the “hypermobility type EDS”. Among 20 obligate heterozygotes for a severely defective *TNXB* allele, 9 of 14 females but no males had hypermobility EDS (69). Dr. Deborah Merke (NIH, Bethesda MD) found that 7% of patients with 21OHD had symptomatic TNX haploinsufficiency (70, 71), and a recent study reported 14% (72); this association is now regarded as a sub-type of 21OHD termed CAH-X (73). Thus, studies of 21OHD and TNX, like their genes, have been linked from the beginning and continue together.

## Author Contributions

WLM assembled the literature, wrote and edited the manuscript, drew the figure, and approved it for publication.

## Funding

WLM’s research was supported in the past by grants from the NIH and the March of Dimes.

## Conflict of Interest

The author declares that the research was conducted in the absence of any commercial or financial relationships that could be construed as a potential conflict of interest.
